# Age‐Associated B Cells: Origins, Regulation, and Tissue‐Specific Pathogenic Contributions in Autoimmune, Metabolic, and Neurological Diseases

**DOI:** 10.1111/imr.70144

**Published:** 2026-08-05

**Authors:** Kira Rubtsova

**Affiliations:** ^1^ Eli Lilly and Company San Diego California USA

## Abstract

Age‐associated B cells (ABCs) are a distinct B cell population characterized by coexpression of T‐bet and CD11c. First described in aged female mice and autoimmune‐prone strains, ABCs are now recognized to be associated with multiple human autoimmune diseases, including systemic lupus erythematosus, rheumatoid arthritis, and multiple sclerosis, where their frequency correlates with disease activity. Their differentiation requires toll‐like receptor (TLR) signaling, IFN‐γ, IL‐21, BCR engagement, with ZEB2 emerging as the nonredundant transcriptional master regulator of ABC identity. Here we review requirements for ABC differentiation, their defining phenotypic and functional features, and their tissue‐specific distribution and pathogenic contributions in diverse inflammatory settings. A central theme is that ABCs are tissue‐homing cells whose contribution extends beyond blood and lymphoid organs. In kidney, inflamed synovium, salivary glands, and adipose tissue, ABCs drive pathology through locally differentiated autoantibody‐secreting cells, stromal activation, antigen presentation, and cytokine production. We further discuss roles for ABCs in neurological diseases, including their enrichment in the cerebrospinal fluid in multiple sclerosis (MS) and their contribution to neuroinflammation in Alzheimer's disease, and highlight the EBV–ABC axis as a mechanism linking viral infection to autoimmunity. In summary, ABCs are multi‐functional, tissue‐adaptable pathogenic effectors whose contributions to human disease extend beyond classical autoimmunity into metabolic dysfunction and neurodegeneration.

## 1 Introduction

B cells play central and diverse roles in the pathogenesis of autoimmune diseases. In addition to autoantibody production, B cells contribute to the autoimmune response via antigen presentation, cytokine secretion, and regulation of T cell responses. High‐dimensional phenotyping and single‐cell profiling have revealed substantial heterogeneity within the B cell compartment and the importance of disease‐associated B cell subsets. Among these, age‐associated B cells (ABCs) have emerged as a distinct population with unique transcriptional, phenotypic, and functional features that position them at the intersection of aging, chronic inflammation, infection, and autoimmunity.

ABCs were initially identified in aged female mice [1, 2] but are also recognized to expand prematurely in a wide range of human autoimmune diseases, including systemic lupus erythematosus (SLE), rheumatoid arthritis (RA), Sjögren's syndrome, multiple sclerosis (MS), and myasthenia gravis (MG), among others [3]. These cells have been originally characterized by high expression of the transcription factor T‐bet and surface markers such as CD11c and CD11b [1, 4]. Functionally, ABCs are hyperresponsive to innate immune stimulation and are primed for rapid differentiation into antibody‐secreting cells, production of pro‐inflammatory cytokines, and antigen presentation.

Accumulating literature suggests that ABCs usually differentiate through extrafollicular differentiation pathways driven by chronic exposure to self‐antigens, intracellular toll‐like receptor (TLR) signaling (particularly TLR7) and inflammatory cytokines such as IFN‐γ and IL‐21 [5, 6, 7]. In multiple autoimmune diseases, ABCs are expanded in parallel with disease activity, enriched in autoreactive clones, and have enhanced antigen presenting ability. Importantly, recent studies have highlighted the heterogeneity within the ABC population, suggesting the existence of multiple transcriptional and functional states which may indicate disease‐ and/or context‐specific features [8].

In this review, we first discuss the history of the discovery of ABCs, providing a framework for how this population has been identified as a distinct B cell subset. We then summarize the current understanding of these cells' defining features and the factors that drive their differentiation. In the last chapters of this review, we focus on the tissue‐specific distribution and functional roles of ABCs in diverse settings. We highlight ABC biology in autoimmune diseases such as lupus, rheumatoid arthritis, and Sjögren's disease. We will also extend to conditions not traditionally considered B cell–driven, including adipose tissue associated metabolic dysfunction, and Alzheimer's disease. By reviewing the findings published across these systems, we aim to provide a view of ABCs as a pathogenic immune cell population which adapts to the tissue environment and contributes to the pathology of autoimmune, as well as nonautoimmune, diseases.

## 2 History of the Discovery of ABCs

ABCs (identified as CD11c + B cells) were first described in two complementary studies published in 2011. Both studies defined a previously unrecognized mature B cell population that accumulated with age in female mice and in autoimmune mouse models. Two studies described the same population from different biological angles; however, together they established ABCs as a previously undescribed, distinct, and physiologically relevant B cell subset.

The first study, by Rubtsov et al. [1] identified ABCs in the context of female‐biased autoimmunity. The authors described a population of CD11c^+^CD11b^+^CD21^−^ B cells that accumulated in aged female mice and in lupus‐like autoimmune‐prone strains. This study also provided early evidence of the presence of human ABCs in aged healthy females and in patients with autoimmune diseases. The data suggested that ABCs were producing higher titers of autoantibodies than follicular B cells upon TLR7 stimulation and depended on TLR7 signaling for their development, providing a mechanistic link to sex bias given the X‐linked nature of *Tlr7*. Collectively, this work established ABCs as a pathogenic, innate‐responsive population enriched in both murine and human autoimmune settings.

In parallel, Hao et al. [2] identified a phenotypically related subset (CD21/35^−^CD23^−^) through unbiased profiling of splenic B cells across aging. In this study, ABCs were demonstrated to accumulate with age, particularly in females, and were defined functionally by poor responses to BCR/CD40 stimulation but strong proliferation in response to TLR7/9 signals. They also exhibited reduced dependence on BAFF, further distinguishing them from conventional follicular and marginal zone B cells. This innate‐skewed responsiveness provided a functional framework for understanding ABC behavior.

Subsequent studies further confirmed that these phenotypes represent the same population, characterized by CD11c expression and TLR‐driven activation, with T‐bet later identified as a key transcriptional regulator [4, 6]. In both mice and humans, ABC‐like cells are now defined by a shared program of innate activation, antigen experience, and association with inflammatory and autoimmune contexts.

Together, these studies established ABCs as a mature, TLR‐responsive B cell subset that accumulates with age, exhibits a female bias in age, and contributes to autoimmune pathology.

## 3 ABC Differentiation: Innate Signals, Cytokines, and Transcriptional Control

ABC differentiation is now understood to require costimulation with innate, antigen‐driven, and cytokine‐mediated stimuli. TLR7, the receptor which senses single‐stranded RNA (ssRNA) and drives MyD88‐dependent activation, plays a dominant role in the differentiation of ABCs. X‐linked expression of TLR7 potentially provides a mechanistic explanation for the female bias observed in ABC accumulation in aged mice [9]. Since *tlr7* is encoded on the X chromosome, females carry two copies of the gene. In females, one of the two X chromosomes is randomly inactivated in each somatic cell during early embryonic development. This process, known as X chromosome inactivation, ensures equivalent gene dosage between XX females and XY males [10]. It has been recently demonstrated that *tlr7* can escape X chromosome inactivation in immune cells, resulting in a substantial fraction of female immune cells that express TLR7 from both X chromosomes simultaneously, resulting in doubling their TLR7 signaling capacity relative to males [11]. Female B cells expressing two copies of TLR7 were shown to have stronger TLR7‐driven responses including greater IgG class switching and plasmablast differentiation, and TLR7 dosage directly controls sex‐specific ABC accumulation in vivo with TLR7 duplication in males being sufficient to override the female ABC bias and drive severe autoimmunity [12].

BCR engagement alone is insufficient to drive ABC differentiation. However, it acts synergistically with TLR signaling, which likely mimics the recognition of self‐derived nucleic acid–containing antigens commonly recognized in many autoimmune diseases. In addition, cytokines contribute to ABC differentiation; thus, IFN‐γ induces T‐bet and primes B cells for TLR responsiveness, while IL‐21 supports extrafollicular differentiation and antibody production [7, 13, 14]. In contrast, IL‐4 antagonizes this pathway, restricting ABC formation to inflammatory environments characterized by IFN‐γ and IL‐21 [13].

Importantly, ABCs arise preferentially from autoreactive B cells that experience chronic low‐level BCR signaling. Persistent engagement with self‐antigen, together with TLR ligands and inflammatory cytokines, drives B cells conversion into ABCs [15]. In vivo, this process is further reinforced by T cell help through CD40–CD40L interactions and cytokine support from T follicular helper (Tfh) and T peripheral helper (Tph) cells, suggesting that ABC development is dependent on T cell help [16, 17]. At the transcriptional level, these signals (BCR, TLR, and cytokines) are consolidated into a stable cell fate by a defined transcriptional program. ZEB2 has recently emerged as a nonredundant master regulator that suppresses germinal center differentiation and enforces key features of ABC identity such as CD11c expression [18, 19]. While factors such as T‐bet, IRF5 [20], and IRF8 [21] shape ABC function, ZEB2 is now recognized to play a central role in driving and maintaining the ABC identity.

## 4 Defining Features of ABCs

ABCs are distinguished from other B cell subsets by a set of phenotypic and functional features. Phenotypically, they are different from both follicular and marginal zone lineages, lacking CD21 and CD23 [1, 2] while expressing markers such as CD11c, T‐bet, and FCRL5 [22], together with high levels of surface receptors involved in antigen presentation and costimulation [23]. Their chemokine receptor profile drives their migration to inflamed peripheral tissues rather than lymphoid follicles [19, 24], indicating their close association with sites of chronic inflammation (discussed in details below). Transcriptionally, ABCs form a distinct and conserved cluster characterized by enrichment of innate sensing, inflammatory, and antigen presentation pathways, coupled with suppression of germinal center and follicular programs [8, 19, 22].

**FIGURE 1:**
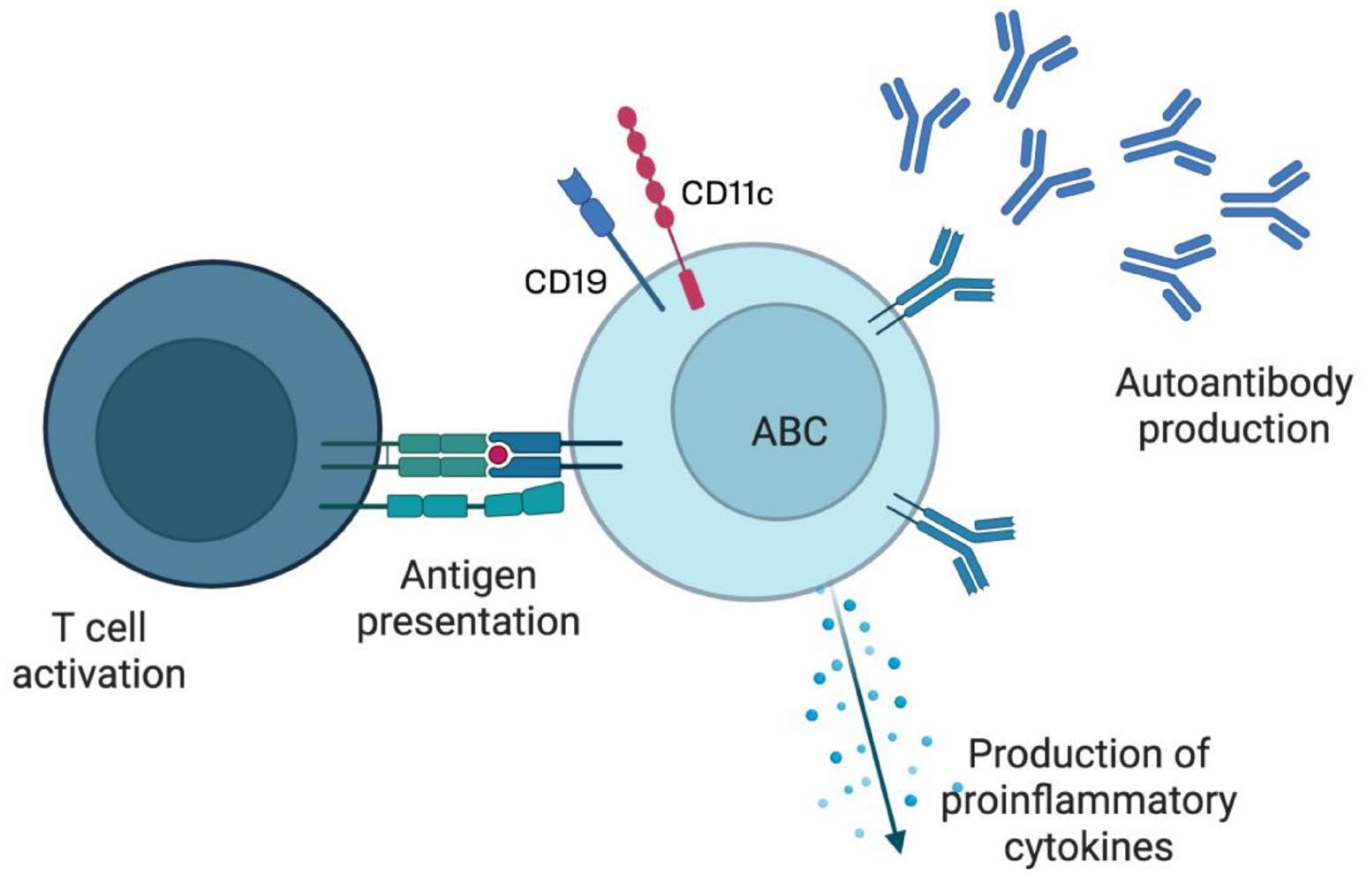
Functional properties of age‐associated B cells (ABCs). ABCs contribute to autoimmune pathology through three major mechanisms. First, they act as potent antigen‐presenting cells, expressing high levels of MHC class II and costimulatory molecules, enabling efficient antigen presentation to CD4^+^ T cells and sustaining autoreactive T cell responses. Second, ABCs can rapidly differentiate through extrafollicular pathways into antibody‐secreting cells producing class‐switched autoantibodies that drive immune complex deposition and organ damage. Third, ABCs secrete pro‐inflammatory cytokines including TNF‐α, IFN‐γ, and IL‐6, amplifying local inflammation independently of antibody production. Created in https://BioRender.com.

Functionally, ABCs exhibit a very distinct pattern of responses to various stimuli when compared to other B cell subsets. Thus, ABCs are hyporesponsive to BCR and CD40 stimulation, which is consistent with their origin from chronically stimulated, possibly anergic precursors. However, ABCs respond robustly to TLR signals and pro‐inflammatory cytokines such as IFN‐γ and IL‐21 [1, 2]. They are also independent of BAFF for survival [2] and preferentially differentiate through an extrafollicular pathway rather than participating in germinal center reactions [17, 25] producing class‐switched antibodies with limited affinity maturation [25]. Beyond antibody production, ABCs act as potent antigen‐presenting cells, sources of inflammatory cytokines, and have been demonstrated to display enhanced phagocytic capacity, further reinforcing their hybrid identity (Figure 1) [19, 23]. Collectively, these properties define ABCs as rapid, innate‐like effector B cells adapted to inflammatory environments, with functions that can drive pathology when chronically engaged by self‐antigen.

## 5 Age‐Associated B Cells in DIfferent Tissues: Distribution, Phenotype, and Pathogenic Roles

During the first years after their discovery, ABCs were investigated mainly in blood and spleen, mostly due to feasibility of sampling. However, defining biological properties of ABCs predicts an inherently tissue‐homing character. Their expression of CXCR3 and CCR2, often combined with the lack of CXCR5 and the high expression levels of integrins including CD11b and CD11c, equips ABCs to leave secondary lymphoid organs and home toward sites of chronic IFN‐γ driven tissue inflammation [19, 24, 25]. Across the last several years, paired blood–tissue sampling, spatial transcriptomics, multiplex immunohistochemistry, and single‐cell RNA sequencing have substantially clarified the tissue presence, as well as characteristics of tissue ABCs, revealing that these cells are not only expanded in the circulation during autoimmunity, but also are present, actively enriched, and in many settings phenotypically and functionally shaped by nonlymphoid tissues that they infiltrate [24, 26, 27].

**FIGURE 2:**
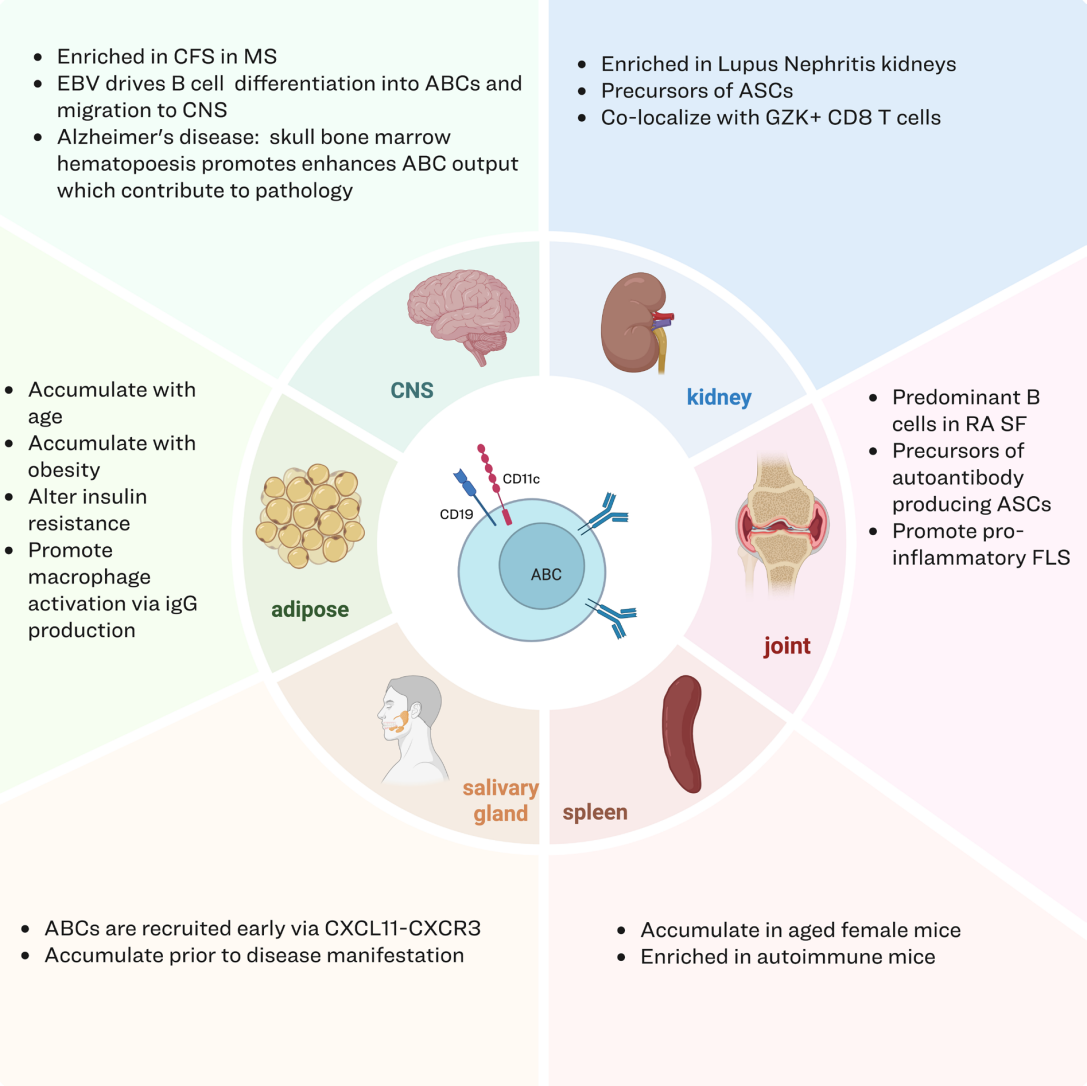
ABCs in lymphoid and nonlymphoid tissues and their organ‐specific pathogenic roles. ABCs accumulate across different tissues where they adopt tissue‐specific phenotypes and contribute to local pathology. Key sites include the kidney in lupus nephritis, synovial fluid and tissue in rheumatoid arthritis, salivary glands in Sjögren's disease, adipose tissue in aging and obesity, and the central nervous system in multiple sclerosis and Alzheimer's disease. In each tissue, ABCs engage distinct but often overlapping pathogenic mechanisms, including local autoantibody production, stromal cell activation, antigen presentation, and inflammatory cytokine secretion. Created in https://BioRender.com.

The distribution, phenotypic adaptation, and pathogenic contributions of ABCs across different diverse tissue compartments, including the kidney, synovium, salivary glands, adipose tissue, and central nervous system, are described in detail below and summarized in Figure 2.

### 5.1 Lymphoid Organs: Spleen and Lymph Nodes

In aged mice, ABCs progressively accumulate in the spleen, becoming a substantial fraction (up to 30%) of all mature splenic B cells in 22 month old females [1, 2]. This accumulation is accelerated in lupus‐prone backgrounds. Thus, in MRL/lpr, NZB/W F1, TLR7‐transgenic, and DEF6/SWAP‐70 double‐knockout mice, splenic ABC expansion occurs before the manifestation of disease, with ABCs reaching high frequencies in young animals [1, 20]. This early expansion supports the concept that ABCs are not simply a consequence of autoimmune pathology but may instead participate in early systemic immune activation that predisposes to autoimmunity by differentiating in the inflamed tissues before disease is clinically apparent.

Splenic ABCs in these autoimmune models are activated and antigen‐experienced, characterized by high MHC‐II and costimulatory molecule expression, evidence of somatic hypermutation and class switching to IgG2c, and CXCR3 expression that eventually drives them to migrate from the spleen toward inflamed peripheral sites [15]. The ZEB2‐dependent transcriptional program of these splenic ABCs enforces their extrafollicular identity by suppressing Bcl6 and Mef2b (the transcription factors required for GC entry) while sustaining CD11c expression and phagocytic capacity [19].

While the great majority of human ABC research has been conducted in peripheral blood, important evidence places ABCs directly within human secondary lymphoid tissues. The most direct human secondary lymphoid organ data comes from the tonsil. Ambegaonkar et al. [28] used human tonsil naïve B cells, memory B cells, and germinal center B cells to systematically model ABC differentiation in vitro, demonstrating that naïve tonsillar B cells preferentially gave rise to T‐bet^+^ atypical B cells (ABCs) under conditions of TLR9 stimulation combined with IFN‐γ and antigen, confirming that human secondary lymphoid organ B cells are competent to generate ABCs in the appropriate microenvironment.

In human spleen, scRNA‐seq datasets have confirmed the presence of T‐bet^+^CD11c^+^ B cell clusters as a discrete population distinct from follicular, marginal zone, and classical memory B cells, which confirms the conservation of splenic ABC identity between mice and humans [29]. A 2025 organ‐based characterization of B cells in SLE patients confirmed that ABCs (including the atypical memory and DN2 populations) are present within the spleen and secondary lymphoid organs of lupus patients as part of a systemic extrafollicular B cell dysregulation, alongside GC‐derived plasma cells and regulatory B cells [29].

### 5.2 Kidney in Lupus Nephritis

Lupus nephritis has provided clear evidence that ABCs can accumulate in nonlymphoid tissues and contribute directly to local autoimmune pathology. The most mechanistically detailed characterization of intrarenal ABCs to date comes from Wu et al. [26] where the authors performed paired scRNA‐seq and BCR/TCR sequencing on sorted B and T cells from kidney biopsies and matched peripheral blood of patients with active lupus nephritis. High infiltration of intrarenal ABCs and antibody‐secreting cells was identified in the B cell compartment. A unique expansion of IGHG4 clones and clonal overlap between ABCs and antibody‐secreting cells (ASCs) was found in kidney‐specific clonotypes, demonstrating a potent extrafollicular B cell response occurring in situ within the nephritic kidney. These data suggest that ABCs serve as local precursors to kidney‐resident antibody‐secreting cells rather than simply being recruited from the periphery. The T cell findings were equally important. In the T cell compartment, granzyme K^+^ (GZMK^+^) CD8^+^ T cells were identified as the dominant kidney‐associated T cell population. Intrarenal GZMK^+^CD8^+^ T cells highly expressed IFN‐γ and displayed strong intercellular communication with ABCs. These two cell subsets largely colocalized within tertiary lymphoid structures (TLS), suggesting that GZMK+CD8+ T cells potentially contributed to ABC differentiation via IFN‐γ and IL‐21 production. This establishes a compelling intrarenal immunopathogenic network: activated by local antigens and stress signals, GZMK^+^CD8^+^ T cells produce IFN‐γ, which drives T‐bet expression and ABC differentiation within the kidney. The resulting ABCs then receive IL‐21 from colocalized Tfh‐like cells to differentiate into locally class‐switched ASCs producing kidney‐depositing autoantibodies.

Multiple reports suggest that anatomical localization of B cells within the kidney is not random. Intrarenal B cells are predominantly located in the tubulointerstitial compartment rather than in glomeruli [30] within the interstitium, and are organized into tertiary lymphoid structures of different developmental complexity. Four organizational levels of intrarenal TLSs have been identified in LN patients, ranging from scattered B cells to highly structured clusters with central follicular dendritic cells [31].

ABCs within the lupus nephritis kidney contribute to pathology through at least three nonredundant mechanisms. First, as direct ASC precursors, intrarenal ABCs generate locally class‐switched IgG‐producing plasma cells whose antibody output deposits as immune complexes in the glomerulus and tubular basement membrane, activating complement and myeloid cells via Fc receptors to drive kidney damage. The clonal specificity of kidney‐derived ABC–ASC pairs for nuclear autoantigens, including anti‐dsDNA and anti‐Sm/RNP specificities, suggest these auto‐antibodies are locally generated by ABC‐ASC differenttaion [26, 32].

Second, as potent antigen‐presenting cells, intrarenal ABCs expressing high levels of MHC‐II and costimulatory molecules can activate resident and infiltrating CD4^+^ T cells within the renal TLS, sustaining local T cell help for continued B cell differentiation and creating a self‐amplifying auto‐immune loop independent of the secondary lymphoid organs [26, 31]. This local antigen presentation function could explain why renal TLS persist and expand even when systemic immune activity is partially controlled by immunosuppression.

Third, ABCs are known to produce pro‐inflammatory cytokines including TNF‐α, IFN‐γ, and IL‐6, and could contribute directly to the pro‐inflammatory cytokine environment of the lupus nephritis kidney by activating tubular epithelial cells.

### 5.3 Synovial Fluid and Synovial Tissue in Rheumatoid Arthritis

The joints of rheumatoid arthritis (RA) patients provide important demonstration that ABCs are not only present but also strongly enriched at sites of inflammation. Studies comparing peripheral blood (PB) and synovial fluid in early drug‐naïve RA show that ABCs are rare in blood but can become the predominant B cell subset in synovial fluid [24]. Phenotypically, synovial fluid ABCs (similar to kidney ABCs) display hallmarks consistent with local activation and tissue retention, including increased activation markers, proliferation signals, antigen presentation features, and altered Fc receptor–like (FcRL) profiles compared with blood ABCs. The notable shift from a minor population in blood to a predominant one in the joint is likely driven by active recruitment. Synovial fibroblasts and macrophages produce CXCL9, CXCL10, and CXCL11, which establish chemokine gradients that attract CXCR3‐expressing ABCs. High CXCR3 expression promotes ABCs' homing to inflamed joints rather than lymphoid organs, explaining their selective accumulation in synovial fluid.

To better understand the relationship between ABCs and other synovial B cell subsets, the scRNA‐seq with paired BCR sequencing has been performed on highly purified CD19^+^ B cells isolated from the synovial tissue of seropositive RA patients [33]. In this study, unsupervised clustering identified 12 distinct B cell subsets, including naïve B cells, memory B cells, and a CD11c^+^ double‐negative 2 (DN2) cluster, the ABC‐equivalent population. BCR lineage analysis directly confirmed clonal relationships between ABCs and ASCs within the synovial tissue, strongly suggesting that ABCs serve as the major precursors of synovial ASCs. While ABCs had previously been suggested as ASC precursors in lupus [34], this study provided the first proven clonal link between these two subsets specifically in RA, establishing that the synovial ABC population serves as a source of locally generated pathogenic autoantibody‐secreting cells in the RA joint.

These findings were independently confirmed and substantially extended by Dunlap et al. [35], who performed single‐cell RNA and paired BCR/TCR repertoire sequencing on synovial tissue and matched peripheral blood samples from 12 seropositive RA patients, providing a comprehensive simultaneous analysis of T and B cell transcriptomes and antigen receptor repertoires across tissue and blood compartments at single‐cell resolution. In this cohort, ABCs and ASCs were also found to be selectively enriched in the synovium relative to peripheral blood and displayed higher somatic hypermutation rates compared to their circulating counterparts, suggesting ongoing local antigen‐driven selection within the joint. Most importantly, BCR lineage analysis directly identified ABCs, memory B cells, and activated B cells as clonally linked to synovial ASCs independently validating in a separate cohort and methodology the ABC–ASC clonal relationship established above, and further demonstrating that this connection is a reproducible and generalizable feature of B cell biology in the RA synovium rather than a cohort‐specific observation. Taken together, these two studies suggest a consistent model in which synovial ABCs play a central role in the local humoral immune response in the RA joint, serving as the clonal precursor to locally differentiating ASCs. Thus ABCs appear to serve as a source of autoantibody production in the inflamed tissue, suggesting that the autoantibodies produced within the RA joint are not exclusively derived from plasma cells recruited from lymphoid organs, but can arise locally from synovial ABCs that have undergone extrafollicular differentiation in situ. Furthermore, synovial tissue studies have additionally demonstrated ongoing somatic hypermutation and class‐switch recombination within TLS of the RA synovium, with FDC‐containing follicles producing autoantibodies independently of new B cell influx from the circulation [36].

The pathogenic relevance of synovial ABCs is further supported by their correlation with clinical disease severity. Across RA cohorts frequency of ABCs in blood, synovial fluid, and synovial tissue positively correlates with disease activity scores [37, 38]. This correlation between ABC burden and disease severity across all three compartments reinforces the interpretation that ABCs are not epiphenomenal bystanders elevated passively during systemic inflammation, but active contributors whose accumulation within the joint drives local pathogenic autoimmune activity.

Beyond their role as clonal precursors to locally differentiating plasma cells, synovial ABCs contribute to joint pathology through a second, entirely distinct and nonantibody‐mediated mechanism: the direct activation of fibroblast‐like synoviocytes (FLS) [37]. This finding is important because it decouples ABC pathogenicity from autoantibody production, establishing that ABCs can drive structural joint damage through stromal cell modulation independently of their capacity to generate autoantibodies. FLSs are the dominant stromal cell type of the synovial lining and the principal effectors of pannus formation, cartilage invasion, and bone erosion in RA [39]. Yet their activation by specific B cell subsets had not been characterized until the ABC–FLS axis was described where coculture of FLS with RA ABCs or ABC‐conditioned medium led to the activation of FLS, coupled with increased production of IL‐6, MMP‐1, MMP‐3, and MMP‐13. Mechanistically, ABC‐conditioned medium–derived TNF‐α promoted upregulation of interferon‐stimulated genes in FLS, with elevated phosphorylation of ERK1/2 and STAT1. Blockade of ERK1/2 and JAK‐STAT1 pathways inhibited ABC‐induced FLS activation, establishing that ABCs contribute to RA pathogenesis by inducing FLS activation via TNF‐α‐mediated ERK1/2 and JAK‐STAT1 signaling [37].

The downstream consequences of this ABC‐FLS axis are directly relevant to the features of RA pathology. Elevated IL‐6 from ABC‐activated FLS amplifies systemic inflammatory features including acute‐phase response with MMP‐1, MMP‐3, and MMP‐13 (matrix metalloproteinases responsible for cartilage degradation [40, 41]), suggesting that ABC‐driven FLS activation directly contributes to the irreversible structural joint destruction. Crucially, this pathway operates entirely independently of autoantibodies, providing a mechanistic explanation for why a subset of patients with aggressive erosive disease maintain relatively modest autoantibody titers [42]. The ABC‐FLS axis can drive joint destruction through cytokine and protease‐mediated stromal activation even in the relative absence of antibody‐mediated effector mechanisms.

The relationship between synovial ABCs and FLS is likely not unidirectional. FLS have been shown to actively support the retention and survival of synovial B cells through contact‐dependent mechanisms involving SDF‐1 and VCAM‐1 facilitating the migration of B cells beneath the synoviocyte layer [43, 44]. While these foundational studies characterized total synovial B cells rather than ABCs specifically, the mechanistic basis for ABC involvement is compelling: ABCs express VLA‐4, the canonical counter‐receptor for VCAM‐1, and CXCR4, the receptor for SDF‐1, suggesting that ABCs can engage the same FLS‐mediated survival niche that sustains memory B cells and plasma cells within the inflamed joint. If confirmed for ABCs specifically, which remains an important open experimental question, this would establish a reciprocal ABC–FLS circuit in which ABCs activate FLS through TNF‐α to produce IL‐6 and matrix‐degrading MMPs [37], while FLS in turn provide stromal niche signals that retain and sustain ABCs within the synovium, creating a self‐amplifying local inflammatory loop independent of continuous ABC recruitment from the circulation. This bidirectional ABC–FLS relationship, operating in parallel with the ABC–ASC axis of local autoantibody production, would position synovial ABCs as dual‐function pathogenic effectors, simultaneously driving humoral autoimmunity through clonal ASC differentiation and stromal inflammation through FLS activation. These two pathogenic axes together could account for both the serological and the structurally destructive features of RA [45].

In summary, ABCs in the RA joint are locally enriched, tissue‐adapted, multi‐effector populations whose frequency correlates with disease activity and whose contributions span the full spectrum of RA pathology, from autoantibody‐mediated immune complex deposition to direct stromal activation driving structural joint destruction.

### 5.4 Salivary Glands in Sjogren's Disease

Salivary glands provide another example of a nonlymphoid tissue where ABCs accumulate and appear to participate as early contributors to the tissue infiltration cascade that precedes and potentially initiates chronic tissue pathology. The Sjögren's disease (SjD) context is important for understanding ABC tissue biology because it allows the temporal sequence of events from chemokine induction to ABC recruitment to lymphocytic focus formation to glandular dysfunction to be mapped in a mouse model.

Characterization of ABC biology in salivary glands comes from Bagavant et al. [46], who used female C57BL/6 mice, a strain that spontaneously develops Sjögren's‐like salivary gland pathology with age, to map the temporal sequence of glandular immune events at different time points. This study demonstrated that at 8 months, prior to the development of lymphocytic foci, gene expression analysis of salivary glands showed significant upregulation of several chemokines involved in immune cell trafficking, including CXCL9, CXCL11, CCL5, and CCL8. At this same timepoint, ABCs expressing CD11b and/or CD11c were already preferentially enriched in the salivary glands compared to spleen and liver. Salivary gland ABCs increased progressively with age and positively correlated with CD4^+^ Tfh cell numbers. By 14 months, well‐organized lymphocytic foci of T and B cells had spontaneously developed, accompanied by high titers of serum autoantibodies, with a subset of aged mice developing salivary gland dysfunction mimicking SjD [46].

The chronological order of these events, chemokine upregulation and ABC enrichment at 8 months, followed by lymphocytic foci formation at 14 months, suggests important mechanistic implications. The upregulation of CXCL11 in the salivary gland stroma before lymphocytic foci are histologically detectable indicates that the aging glandular epithelium establishes a pro‐inflammatory chemokine gradient that actively recruits CXCR3^+^ ABCs prior to overt immune infiltration. Thus ABCs in the salivary gland arrive at the earliest stages of tissue inflammation, rather than accumulating as consequences of established ectopic lymphoid structures, suggesting they may participate in initiating and organizing the lymphocytic infiltrate rather than responding to it. The selective enrichment of ABCs in the salivary gland relative to spleen and liver at this early timepoint further indicates that gland‐specific recruitment or retention signals drive their preferential accumulation in this tissue.

In addition to mouse data, Nishida et al. [47] has recently provided the first direct evidence of ABCs accumulation within human salivary gland biopsies from SjD patients. T‐bet^+^CD20^+^ ABCs were detected infiltrating the labial salivary glands of 44 SjD patients, particularly in individuals in their 40s–60s, but were rare in non‐SjD controls establishing that salivary gland ABC infiltration is specific to the autoimmune context rather than a consequence of aging alone. Authors also demonstrated significant increase in ABCs in the salivary glands of SjD model mice, but not in age‐matched control mice or young SjD model mice, confirming that ABC accumulation in the gland is disease‐driven and age‐dependent rather than a universal feature of the tissue. Mechanistically, CD4^+^ Tfh‐like cells were identified within the salivary gland as the major local source of IL‐21, which together with IFN‐γ drove T‐bet upregulation and ABC differentiation within the gland. Another important finding was that ABCs themselves produced IFN‐γ creating an autocrine loop that sustains their own expansion within the gland independently of T cells.

Collectively, the evidence from both mouse models and human salivary gland biopsies suggest that ABCs serve as early and active participants in SjD pathogenesis recruited to the gland by CXCL11–CXCR3 chemokine gradients and sustained locally by autocrine IFN‐γ and Tfh‐derived IL‐21 [47]. Based on these findings, a hypothesis for ABC‐driven SjD pathogenesis emerges: early ABC recruitment establishes an initial IFN‐γ–rich inflammatory niche within the gland that amplifies CXCR3 ligand production, drawing in further waves of CXCR3^+^ T cells and ABCs in a self‐reinforcing recruitment loop. Once established, glandular ABCs serve as potent antigen‐presenting cells that activate local autoreactive CD4^+^ T cells, providing specific environment for lymphocytic focus organization, and differentiate into ASCs producing autoantibodies locally within the gland. Whether ABCs are causal drivers of this progression or amplifiers of a preexisting autoimmune response remains to be directly tested, but their early appearance, tissue‐specific enrichment, and pathogenic profile makes them strong candidates for therapeutic targeting at the earliest stages of Sjögren's disease.

### 5.5 Adipose Tissue: Obesity and Metabolic Disorders

Adipose tissue is increasingly recognized as an immunologically active organ, harboring diverse immune cell populations including macrophages, innate lymphoid cells, regulatory T cells, and B cells whose composition and activation state are profoundly remodeled by aging and obesity and directly shape systemic metabolic outcomes [48]. Within this immune landscape, B cells occupy a particular niche in visceral adipose tissue, where they accumulate within fat‐associated lymphoid clusters (FALCs) [49], specialized ectopic lymphoid structures that expand with age and metabolic stress, and contribute to adipose inflammation through cytokine production, T cell activation, and local immunoglobulin secretion. Among adipose B cell subsets, aged adipose B cells (AABs) have emerged as a disease‐relevant population. In aged visceral adipose tissue, AABs are found within FALCs, and their accumulation correlated directly with impaired metabolic homeostasis including defective lipolysis and impaired thermoregulation [50]. Transcriptomic profiling has revealed that adipose AABs are distinct from splenic ABCs not only at the transcriptional level but also at the surface phenotype level, lacking CD11c expression that characterizes splenic ABCs, while sharing the CD21^−^CD23^−^ nonfollicular identity. This suggests that the adipose tissue microenvironment generates an ABC‐related but transcriptionally and phenotypically distinct B cell population, shaped by local innate signals including NLRP3 and IL‐1 rather than the IFN‐γ/TLR7 axis that drives splenic ABC formation. The FALC formation and AAB expansion have been demonstrated to be dependent on NLRP3 inflammasome activity providing a direct mechanistic link between age‐associated innate inflammation and adipose immune remodeling, positioning the inflammasome as an upstream regulator that promotes the accumulation of pathogenic B cells within the aging fat [50]. The relationship between AABs and splenic ABCs and whether they represent the same lineage shaped by different tissue environments or are distinct B cell populations that share only some phenotypic similarities remains unclear.

While the relationship between AABs and ABCs remains unresolved, obesity drives the accumulation of a T‐bet^+^CD11c^+^ ABC‐like population in adipose tissue that phenotypically resembles splenic ABCs more closely than AABs. In both high‐fat diet (HFD) mouse models and obese humans, T‐bet^+^CD11c^+^ B cells accumulate in adipose tissue and their frequency correlates positively with BMI [51]. Unlike the aging‐associated AAB expansion driven by NLRP3 and IL‐1, obesity‐associated ABC accumulation has been demonstrated to depend on invariant NKT (iNKT) cells and TLR7 stimulation, potentially indicating that distinct upstream innate signals converge on the same ABC‐like phenotype through different pathways depending on whether the inflammatory context is aging or metabolic stress [51].

The pathogenic consequences of this obesity‐driven ABC expansion were identified in this study. ABC‐like B cells in obese adipose tissue produce class‐switched IgG antibodies that bind to apoptotic adipocytes and form immune complexes, which in turn are recognized by Fc receptors on adipose tissue macrophages. This triggers macrophage activation, TNF‐α and IL‐6 production, and downstream impairment of insulin signaling, directly linking ABC‐derived antibody output to the insulin resistance and metabolic dysfunction characteristic of obesity‐associated inflammation. Moreover, B cell‐specific deletion of T‐bet in mice abrogated this IgG production, reduced inflammatory macrophage accumulation in adipose tissue, and ameliorated metabolic disease, while adoptive transfer of class‐switched IgG from obese wild‐type mice into T‐bet‐deficient hosts restored metabolic dysfunction, establishing ABC‐derived IgG as the causal effector rather in this process [51].

Human adipose tissue studies complement the mouse work by identifying DN B cells with CD11c surface expression and T‐bet mRNA upregulation in the subcutaneous adipose tissue of obese individuals, with frequencies correlating positively with BMI. Importantly, adipose B cells in these settings can produce fat‐reactive IgG antibodies even without exogenous stimulation, consistent with ongoing local immune activation [52, 53].

The relationship between BMI, B cell biology, and humoral immunity remains a significantly underappreciated dimension of metabolic disease, and has received less attention than the roles of macrophages and T cells in adipose tissue inflammation. Obesity has been demonstrated to alter B cell subset composition, differentiation, and function both systemically and within adipose tissue, with these changes contributing to immune dysregulation as well as to impaired responses to novel pathogens and vaccines [54]. The inflammatory B cell landscape in obesity is characterized not only by ABC‐like cell expansion but by a broader shift in the balance of pro‐ and anti‐inflammatory subsets: B cells from obese individuals show higher production of IL‐6 and TNF‐α and lower production of IL‐10 compared to lean subjects upon stimulation. In addition a reduction in peripheral transitional B cells and their IL‐10 production was demonstrated suggesting that obesity simultaneously expands pathogenic and depletes regulatory B cell subsets [54].

A review by Frasca et al. [55] proposed that obesity should be considered a biomarker of accelerated B cell aging with similarities between obese and elderly individuals in peripheral B cell composition, antibody responses, and autoimmune antibody secretion suggesting that obesity and aging share cellular and molecular pathways that converge on humoral immune dysfunction. Both conditions are characterized by an expansion of pro‐inflammatory DN/ABC‐like B cells expressing CD11c and T‐bet, a reduction in naive and transitional B cells, decreased responses to vaccination, and increased spontaneous production of autoimmune IgG antibodies [52, 53]. At the molecular level, both aging and obesity are associated with elevated serum levels of pro‐inflammatory cytokines, including TNF‐α, IL‐6, and IL‐1β, and with reduced IL‐10 production by B cells [54]. The functional consequence is also similar in both settings: impaired protective antibody responses to vaccines and infections alongside enhanced production of pathogenic autoimmune antibodies reflecting similar changes in B cell subset balance.

A key question is whether the obesity‐driven inflammatory B cell phenotype is fixed or reversible. Frasca et al. [56] addressed this directly by following obese women through a voluntary weight loss program that reduced BMI by 10%–34% over 12 months. After weight loss, B cell function improved with higher vaccine‐specific antibody responses and lower autoimmune antibody output which is more similar to the profile seen in lean adults [56]. This suggests that the ABC‐like inflammatory state in obesity is not permanent but is actively sustained by ongoing metabolic signals, and that reducing adiposity can at least partially reset the pathogenic humoral immune environment. Whether this improvement reflects a direct reduction in ABC frequency, a phenotypic shift within the ABC compartment, or broader changes in B cell subset balance remains to be determined but the findings raise the possibility that weight loss, together with B cell–targeted therapies, could form part of a strategy to normalize obesity‐associated humoral immune dysfunction.

Additional clinical evidence for the BMI–humoral immunity link comes from studies of vaccine responses and infection outcomes indicating elevated BMI reduces the humoral response to SARS‐CoV‐2 infection, with impaired memory B cell generation being a central mechanism underlying the increased rates of reinfection and suboptimal neutralizing antibody responses observed in individuals with obesity [57].

Thus adipose tissue emerges as a tissue context where ABC biology extends beyond classical autoimmunity into the metabolic disease. Whether driven by aging through the NLRP3 inflammasome or by obesity through iNKT cell and TLR7 signaling, adipose ABCs result in a common pathogenic output: class‐switched IgG production, macrophage activation, and impaired metabolic homeostasis, that directly links this B cell subset to insulin resistance, adipose inflammation, and defective humoral immunity. The human data confirm that these pathways exists in obese individuals and correlates with BMI. Targeting ABCs in the adipose context may help to simultaneously addresses metabolic dysfunction and the humoral immune defects that make obesity a risk factor for poor vaccine responses and infection outcomes.

### 5.6 Central Nervous System: Multiple Sclerosis and EBV Link

Multiple sclerosis (MS) represents one of the most studied CNS contexts for ABC biology, and a rapidly growing body of evidence now suggests that ABCs/atypical memory B cells are active participants in MS pathogenesis. ABCs are present in the cerebrospinal fluid (CSF) at disproportionately higher frequencies than in blood, associated with early and active disease, and linked to the mechanisms driving CNS inflammation through both antibody‐dependent and antibody‐independent pathways.

Claes et al. [58] provided the first direct characterization of ABCs in MS, demonstrating by paired flow cytometry of blood and CSF that ABCs (defined as CD21^−^/ˡ°ʷ B cells) with pro‐inflammatory characteristics were selectively enriched in the CSF relative to paired peripheral blood. CD21ˡ°ʷ ABCs frequencies substantially were higher in the CSF (amounting up to 60% of B cells) than in blood (up to 20% of B cells). Moreover, ABCs were expanded to a greater extent in a subset of MS patients with a more active inflammatory disease profile [58].

This observation has been further confirmed and substantially extended by El Mahdaoui et al. [59] who characterized CD11c^+^ B cells across 155 participants using paired blood–CSF flow cytometry. This study demonstrated that ABCs were enriched in CSF, produced pro‐inflammatory cytokines, and were partially resistance to anti‐CD20 depletion [59]. Additional study has further confirmed by single‐cell profiling of CSF of 139 subjects across multiple neurological diseases confirmed that ABCs defined by elevated TBX21, ITGAX, and FCRL5 expression were enriched in MS CSF versus healthy controls. This enrichment of ABCs was specific for MS and not other neurological diseases examined in the same study [60].

In contrast to the well‐documented ABC enrichment in the CSF, direct identification of ABCs within human MS CNS parenchyma or meningeal tissue is currently absent from the literature. To the best of our knowledge, no published study has formally characterized T‐bet^+^CD11c^+^ ABCs within human MS brain lesions or meningeal ectopic lymphoid structures. The closest evidence comes from experimental models: in EAE, ABCs are elevated within the CNS and functionally required for latent gammaherpesvirus‐enhanced CNS autoimmune disease [61], and B cell‐specific deletion of ODC1 (the rate‐limiting enzyme of polyamine biosynthesis) drives expansion of CD11c^+^CD21/35^−^CD23^−^IgD^−^ ABCs specifically in the dural meninges, producing MOG‐reactive IgG in the brain and worsening EAE severity [62]. Whether these experimental findings translate to human MS meningeal tissue remains undemonstrated and represents an important open question in the field.

Collectively, ABCs in MS are selectively enriched in the CSF well beyond their blood frequencies, associated with active and early disease, partially resistant to anti‐CD20 depletion, and functionally capable of driving intrathecal inflammation.

Several emerging publications now position ABCs as a link between Epstein–Barr Virus (EBV) infection and MS pathogenesis. Thus in vitro, EBV infection of primary human B cells rapidly generates FCRL4^+^TBX21^+^ atypical memory B cells with pro‐inflammatory and innate immune gene signatures, and both de novo infection and established EBV latency sustain this ABC‐like state [63, 64]. In early MS patients, scRNA‐seq of peripheral B cells showed that ABCs carry EBV‐associated transcriptional signatures, including upregulation of CXCR3, PD‐L1, and PD‐L2, suggesting that EBV primes B cells toward the ABC state [65]. Interestingly, the same group demonstrated in a separate study in SLE that EBV^+^ B cells directly acquire the ABC phenotype expressing T‐bet, ZEB2, CD11c, and FCRL5, and function as pro‐inflammatory antigen‐presenting cells that activate T cells [66]. Similarly, in the MS context, EBV was shown to infect autoreactive B cells which recognize CNS antigens and to drive them into acquiring a pro‐inflammatory APC transcriptional program [67], suggesting that EBV‐driven ABC induction is not disease‐specific but may represent a general mechanism by which EBV converts autoreactive B cells into ABcs across multiple autoimmune diseases.

The most compelling experimental evidence linking EBV to ABC‐mediated MS initiation comes from Läderach et al. [68], who developed a humanized mouse model carrying the principal MS‐risk allele HLA‐DRB1*15:01 and infected these mice with EBV. EBV infection expanded oligoclonal T‐bet^+^CXCR3^+^ ABCs in the blood, spleen, and brain, with shared B cell receptor clones across compartments indicating directed trafficking rather than de novo local expansion. Further, brain imaging revealed that T‐bet^+^CXCR3^+^ B cells localized to submeningeal and perivascular niches, where they recruited T cells via B cell‐derived CXCR3‐ligand chemokines CXCL9 and CXCL10, together with CCL3, CCL4, and CCL5. Comigrating T cell populations included effector memory CD8^+^ T cells and CD4^+^ Th1 and Th17 cells, the T cell subsets that drive CNS demyelination and inflammation in MS. Crucially, EBV directly imprinted this promigratory ABC state: in vitro infection of sorted human B cells rapidly induced T‐bet and favored the outgrowth of CXCR3‐positive lymphoblastoid cell lines. T‐bet^+^CXCR3^+^ ABCs could colonize submeningeal brain regions in the absence of other lymphocytes, and depletion of B cells with rituximab or blocking of CXCR3 significantly decreased lymphocyte infiltration into the CNS providing the first direct experimental evidence that targeting ABCs or their CXCR3‐mediated homing could prevent the T cell microinvasion that initiates MS. These findings were independently supported by an earlier study by van Langelaar et al. [69], which demonstrated induction of brain‐infiltrating T‐bet^+^ B cells in MS patients providing the first human correlate of the ABC CNS‐homing phenomenon that was later mechanistically explained in the EBV context.

Together these studies suggest that EBV infection drives the differentiation of autoreactive B cells into ABC‐like, CXCR3^+^ CNS‐homing effectors that initiate neuroinflammation.

### 5.7 Central Nervous System: Alzheimer's Disease

The role of B cells in Alzheimer's disease (AD) has received substantially less attention than that of microglia, astrocytes, and T cells. However, emerging evidences suggest that B cells serve as active contributors to AD neuroinflammation with both protective and pathogenic roles, influencing neurodegeneration through antibody‐mediated clearance of toxic aggregates and inflammatory activation. At the systemic level, B cell subset composition is progressively altered in AD, with longitudinal profiling demonstrating that B cell profile and receptor repertoire are associated with the progression of AD, and that expanded B cells hyperactivate iPSC‐derived microglia with loss‐of‐function for Aβ clearance [70]. Therapeutic B cell depletion in AD mouse models has been reported to reverse disease progression [71], while specific B cell subsets producing IL‐35 demonstrate neuroprotective effects [72] suggesting that the B cell compartment in AD is not uniformly pathogenic but functionally heterogeneous.

Within this broader B cell landscape, ABCs have now emerged as a specific pathogenic subset of particular relevance to AD. A major recent advance comes from Zhang et al. [73] who identified a bone marrow–brain ABC axis in Alzheimer's disease using a combined approach of human imaging and mouse model experiments [73]. On the human side, PET/CT scanning of AD patients revealed Aβ deposits within the skull bone marrow demonstrating that Aβ accumulates outside the brain in a compartment directly adjacent to CNS‐draining lymphatics. On the mouse side, using 5 × FAD and APP/PS1 models, the authors showed that skull bone marrow Aβ accumulation precedes substantial cerebral Aβ deposits, and that this extra‐cerebral Aβ deposition drives enhanced B lymphopoiesis, specifically increased in ABCs, within the skull bone marrow. In vivo cell tracking further demonstrated that these bone marrow‐derived ABCs migrate into the brain parenchyma, where they aggravate Aβ pathology and enhance microglial reactivity. Mechanistically, Aβ was shown to promote this B cell expansion through IL‐6 signaling, and blockade of IL‐6 receptor with tocilizumab reduced ABC numbers in the skull bone marrow and brain parenchyma and attenuated Aβ pathology in the mouse models. Whether the human skull bone marrow Aβ deposits similarly drive ABC expansion and brain infiltration in AD patients remains to be directly tested.

Together these data suggest that ABCs may play a role at the intersection of aging immunity and neurodegeneration. This raises a broader question: does age‐associated accumulation of ABCs represent a systemic driver of organ inflammation across multiple aging diseases, with tissue‐specific triggers—such as Aβ in AD, EBV in MS, or TLR7 ligands in SLE determining where disease ultimately develops?

## 6 Summary: Emerging Principles of Tissue Distribution of ABCs

The tissue distribution data reviewing secondary lymphoid organs, kidney, synovium, salivary glands, adipose tissue, and the central nervous system highlight several overarching principles that collectively define how ABCs should be understood as a cell type (Table 1)

1. ABCs are inherently tissue‐homing cells whose biology cannot be fully understood from blood alone. Their chemokine receptor profile (CXCR3^+^CCR2^+^CXCR5^−^) equips them to leave secondary lymphoid organs and home selectively toward sites of chronic IFN‐γ–driven inflammation rather than lymphoid follicles [19, 24]. The consistent finding that ABCs are disproportionately enriched at tissue sites relative to blood (most dramatically in synovial fluid, CSF, and salivary glands) confirms that blood ABC frequencies, while clinically informative and disease‐correlated, are an imperfect surrogate for the ABC biology that drives tissue pathology. The direct implication is that mechanistic understanding and therapeutic targeting of ABCs requires tissue‐level characterization, not blood profiling alone.
2. Tissue environments can actively remodel ABC phenotype, function, and identity. This is one of the most important conceptual contributions of the multi‐tissue literature. Adipose AABs are tdistinct from splenic ABCs, imprinted by NLRP3 and IL‐1 signals rather than the IFN‐γ/TLR7 axis that drives splenic ABCs [50]. Synovial fluid ABCs display a shifted FcRL balance, higher proliferative activity, and altered costimulatory profiles compared to their blood counterparts [24]. Intrarenal ABCs form clonal connections to kidney‐specific ASCs that are not mirrored in paired blood samples [26]. Salivary gland ABCs develop an autocrine IFN‐γ loop not described at other sites [47]. Together, these findings argue against treating circulating ABCs as surrogates for tissue ABC biology, each tissue niche imprints a distinct functional state on what is phenotypically the same cell type, with potentially significant consequences for their pathogenic contributions and therapeutic vulnerabilities.
3. TLS formation is a recurring and mechanistically important theme, but its consequences are context‐dependent. Across kidney, salivary glands, synovium, and CNS meninges, ABC accumulation is consistently associated with TLS formation, suggesting that ABCs are not attracted to established TLS but rather actively participate in their organization and maintenance. Thus in the nephritic kidney, synovium, and salivary glands, ABC‐associated TLS directly correlate with tissue damage, disease activity, and poor therapeutic responses [32, 37, 46].
4. The EBV–ABC axis in MS: ABCs as cellular mediators of infection‐triggered autoimmunity. The convergent evidence from SoRelle et al. [65], Läderach et al. [68], and Younis/Robinson et al. [68] establishes that EBV infection drives B cells toward an ABC‐like T‐bet^+^CXCR3^+^ state that homes to the CNS and orchestrates T cell infiltration positioning ABCs as initiators of autoimmune disease in a specific infectious context. This principle may extend beyond MS: if latent infections broadly reprogram B cells toward ABC states, then the tissue distribution and pathogenic consequences of ABCs may in part reflect the history of infectious exposures rather than solely the local inflammatory environment.
5. ABCs in the context of aging extend well beyond classical autoimmunity into neurodegeneration and metabolic disease. The skull bone marrow‐brain ABC axis in Alzheimer's disease [73], the NLRP3‐dependent adipose ABC expansion in aging [50] collectively suggest that ABCs represent a systemic cellular effector of inflammaging, generated in increasing quantities with age through converging hematopoietic and inflammatory mechanisms, then migrating to multiple organ systems where tissue‐specific triggers determine the nature and severity of their local pathogenic effects. Whether age‐associated ABC expansion represents a fundamental driver of multi‐organ aging pathology, or whether it is merely correlated with the inflammation associated with aging, remains one of the open questions.

**TABLE 1:** Emerging principles from the multi‐tissue analysis of ABCs.

Principle	Core finding	Key tissue evidence	Key references
Tissue‐homing	CXCR3 + CCR2 + CXCR5− profile directs ABCs toward IFN‐γ‐rich inflamed tissues rather than lymphoid follicles. Blood ABC frequencies are imperfect surrogates for tissue ABC biology.	Synovial fluid (RA): ABCs are the predominant B cell subset CSF (MS): strongly enriched vs. blood Salivary glands (SjD): ABCs arrive before lymphocytic foci form	Vidal‐Pedrola et al. [24] Claes et al. [58] Dai et al. [19]
ABC identity is influenced by tissue	Each tissue niche imprints a distinct transcriptional and functional state on ABCs despite a shared surface phenotype with circulating ABCs	Adipose ABCs: transcriptionally distinct from splenic ABCs Synovial ABCs: shifted FcRL expression, enhanced Ki‐67+ Intrarenal ABCs: unique clonal connections to kidney‐specific ASCs	Camell et al. [50] Vidal‐Pedrola et al. [24] Wu et al. [26] Nishida et al. [47]
TLS formation	ABC accumulation drives TLS formation across multiple tissues resulting in pathogenic outcomes	Kidney (LN): TLS correlate with disease severity and poor treatment response Synovium (RA): TLS support local autoantibody production Meningeal TLS (EAE): ABC‐like B cells worsen CNS disease	Bagavant et al. [46] Chang et al. [32] Qin et al. [37] Zurawski et al. [62]
EBV‐ABC axis in infection‐triggered autoimmunity	EBV drives B cells toward a T‐bet+CXCR3+ ABC state with CNS‐homing capacity, positioning ABCs as cellular mediators linking viral infection to MS initiation.	EBV‐infected mice: ABCs colonize submeningeal brain regions and recruit T cells Early MS patients: EBV‐associated transcriptional signatures enriched in ABCs	Läderach et al. [68] SoRelle et al. [65] Younis et al. [67]
ABCs as effectors of inflammaging	Age‐associated ABC expansion represents a systemic driver of multi‐organ inflammation. Tissue‐specific triggers select the organ where ABC‐driven pathology is most prominent.	AD: Aβ in skull bone marrow drives ABC generation → brain infiltration → microglial reactivity Aged adipose: NLRP3 drives FALC expansion and AAB accumulation	Zhang et al. [73] Camell et al. [50]

Abbreviations: ABC, age‐associated B cell; ACPA, anti‐citrullinated protein antibody; AD, Alzheimer's disease; AKI, acute kidney injury; APC, antigen‐presenting cell; ASC, antibody‐secreting cell; CKD, chronic kidney disease; CNS, central nervous system; CSF, cerebrospinal fluid; EAE, experimental autoimmune encephalomyelitis; EBV, Epstein–Barr virus; FALC, fat‐associated lymphoid cluster; FcRL, Fc receptor‐like; GC, germinal center; IFN‐γ, interferon‐gamma; LN, lupus nephritis; MS, multiple sclerosis; NLRP3, NLR family pyrin domain‐containing protein 3; RA, rheumatoid arthritis; SAT, senescence‐associated T cell; SjD, Sjögren's disease; TLR, Toll‐like receptor; TLS, tertiary lymphoid structure.

Taken together, the multi‐tissue ABC literature supports a following model: ABCs are generated systemically in response to innate and adaptive inflammatory signals, equipped by their chemokine receptor profile for tissue homing, and then locally shaped by the tissue microenvironment into functionally distinct effector states. Their pathogenic contributions, such as autoantibody production, stromal activation, TLS organization, antigen presentation, and cytokine‐mediated tissue injury, vary by organ but share the common thread of sustaining and amplifying local inflammation. How ABCs are shaped by, and in turn shape, the tissues they localize in therefore emerges as one of the important questions in understanding their contribution to various human diseases.

## Conflicts of Interest

Author is an employee of Eli Lilly and Company.

## Data Availability

Data sharing not applicable to this article as no datasets were generated or analyzed during the current study.
